# An Artificial Intelligence–Based, Personalized Smartphone App to Improve Childhood Immunization Coverage and Timelines Among Children in Pakistan: Protocol for a Randomized Controlled Trial

**DOI:** 10.2196/22996

**Published:** 2020-12-04

**Authors:** Abdul Momin Kazi, Saad Ahmed Qazi, Sadori Khawaja, Nazia Ahsan, Rao Moueed Ahmed, Fareeha Sameen, Muhammad Ayub Khan Mughal, Muhammad Saqib, Sikander Ali, Hussain Kaleemuddin, Yasir Rauf, Mehreen Raza, Saima Jamal, Munir Abbasi, Lampros K Stergioulas

**Affiliations:** 1 Department of Pediatrics and Child Health Aga Khan University Karachi Pakistan; 2 Department of Electrical Engineering NED University of Engineering and Technology Karachi Pakistan; 3 Neurocomputation Lab National Centre of Artificial Intelligence Karachi Pakistan; 4 Department of Civil Engineering NED University of Engineering and Technology Karachi Pakistan; 5 Faculty of Electrical and Computer Engineering NED University of Engineering and Technology Karachi Pakistan; 6 Pharmacy Services Aga Khan University Karachi Pakistan; 7 Surrey Business School University of Surrey Guildford Surrey United Kingdom

**Keywords:** artificial intelligence, AI, routine childhood immunization, EPI, LMICs, mHealth, Pakistan, personalized messages, routine immunization, smartphone apps, vaccine-preventable illnesses

## Abstract

**Background:**

The immunization uptake rates in Pakistan are much lower than desired. Major reasons include lack of awareness, parental forgetfulness regarding schedules, and misinformation regarding vaccines. In light of the COVID-19 pandemic and distancing measures, routine childhood immunization (RCI) coverage has been adversely affected, as caregivers avoid tertiary care hospitals or primary health centers. Innovative and cost-effective measures must be taken to understand and deal with the issue of low immunization rates. However, only a few smartphone-based interventions have been carried out in low- and middle-income countries (LMICs) to improve RCI.

**Objective:**

The primary objectives of this study are to evaluate whether a personalized mobile app can improve children’s on-time visits at 10 and 14 weeks of age for RCI as compared with standard care and to determine whether an artificial intelligence model can be incorporated into the app. Secondary objectives are to determine the perceptions and attitudes of caregivers regarding childhood vaccinations and to understand the factors that might influence the effect of a mobile phone–based app on vaccination improvement.

**Methods:**

A mixed methods randomized controlled trial was designed with intervention and control arms. The study will be conducted at the Aga Khan University Hospital vaccination center. Caregivers of newborns or infants visiting the center for their children’s 6-week vaccination will be recruited. The intervention arm will have access to a smartphone app with text, voice, video, and pictorial messages regarding RCI. This app will be developed based on the findings of the pretrial qualitative component of the study, in addition to *no-show* study findings, which will explore caregivers’ perceptions about RCI and a mobile phone–based app in improving RCI coverage.

**Results:**

Pretrial qualitative in-depth interviews were conducted in February 2020. Enrollment of study participants for the randomized controlled trial is in process. Study exit interviews will be conducted at the 14-week immunization visits, provided the caregivers visit the immunization facility at that time, or over the phone when the children are 18 weeks of age.

**Conclusions:**

This study will generate useful insights into the feasibility, acceptability, and usability of an Android-based smartphone app for improving RCI in Pakistan and in LMICs.

**Trial Registration:**

ClinicalTrials.gov NCT04449107; https://clinicaltrials.gov/ct2/show/NCT04449107

**International Registered Report Identifier (IRRID):**

DERR1-10.2196/22996

## Introduction

### Background

Pakistan has an under-5 mortality rate of 69.3 per 1000 live births [1], and is one of the five countries that contributed to half of the under-5 deaths in 2018, with 70% of the deaths being attributed to infectious diseases [2,3]. Pakistan ranks third among countries with the lowest immunization rates for diphtheria-tetanus-pertussis (DTP)–containing vaccine; 40% of children not vaccinated with a 3-dose series of DTP vaccine in 2015 belonged to Pakistan [4]. The low vaccine coverage has led to a number of challenges, including uninterrupted polio transmission and measles outbreaks [5]. In 2013 alone, a measles outbreak resulted in 14,000 cases with 306 deaths [6].

According to the nationwide 2017-2018 Pakistan Demographic and Health Survey (PDHS), 66% of children between the ages of 12 and 23 months had all received the basic vaccines, but only half of the children had age-appropriate vaccines [7]. The yearly PDHS surveys since 1990 have consistently shown that the coverage for age-specific vaccines falls as children grow older, with the highest coverage being seen with the birth vaccine, bacillus Calmette Guérin (BCG) (85%), and the lowest coverage being seen for the 9-month measles-1 vaccine (61%) [8]. In another study, the rate of timely BCG vaccination was 89.3%, with the measles-2 vaccine, given at 15 months of age, having a drop of 2.3% [9]. Low rates of age-appropriate vaccination, even with relatively higher rates of late immunizations, can be extremely detrimental for children as they prolong the duration for which they are vulnerable to illnesses [10].

The barriers preventing widespread immunization are multifaceted, including lack of financing, lack of good governance, supply chain interruption, and lack of awareness in the local population [8]. A case study on the Expanded Programme on Immunization (EPI) in Pakistan considered community misperceptions and misinformation to be one of the most important issues; the program recommended addressing misinformation while taking the local context into account, as the perceptions can vary from region to region and blanket communication may not resonate with all of the communities [11]. Losing vaccination cards is another major reason for low uptake, as many families rely on vaccination cards to know when it is time for the next vaccine. In one study performed on 10,000 caregivers, 76.9% did not have vaccination cards or were missing some data regarding their vaccination status [10]. With a further decrease in immunization rates due to the COVID-19 pandemic, vaccination coverage has plummeted to an all-time low due to disruption in vaccination services and demand, causing new outbreaks of previously controlled diseases, such as new polio strains, measles, and rubella [12]. Therefore, innovative and cost-effective measures need to be taken to understand and address the causes of low immunization rates.

Mobile phones provide an innovative and cost-effective public health intervention and the number of cellular phone subscribers in Pakistan has been steadily growing, with 166 million subscribers as of May 2020 and a tele-density of 78% [13]. Mobile apps and social media have been shown to be effective in various public health programs in developed countries [14-16]. However, the data from low- and middle-income countries (LMICs) are limited on the use of mobile phone–based interventions for improving routine childhood immunization (RCI) coverage; however, a few studies have shown significant promise, even when no additional financial incentives were provided to participants [17]. Most of these studies have used one-way reminder SMS text messages as the study intervention and a few used different forms of messages, such as two-way messages, educational messages, or personalized messages addressing the barrier specific to the recipient [18-23]. There is a need to invest our focus into research that explores the use of mobile phone technology and the design of robust interventions to better understand the utility of these gadgets to bring about a change in the traditional health system.

### Study Objectives

#### Primary Objectives

Our first objective is to evaluate whether a personalized mobile app can improve on-time visits at 10 and 14 weeks of age for RCIs as compared with standard care. Our second objective is to evaluate whether an artificial intelligence (AI)–based model can be incorporated into the app to improve RCI coverage and behavior change.

#### Secondary Objectives

Our secondary objectives are to learn about the perceptions and attitudes of caregivers regarding childhood vaccinations and to find out about factors that might influence the use of mobile phone–based apps for improvement in vaccination coverage.

## Methods

### Target Population

Our target population is caregivers of newborns or infants visiting the Aga Khan University Hospital (AKUH) vaccination center for their children’s 6-week vaccination and whose children are due for their RCIs at ages 10 and 14 weeks, according to Pakistan’s EPI schedule.

### Study Goal

The study goal is to conduct a mixed methods study to assess the acceptability and usability of a personalized, smartphone-based, behavioral intervention to improve vaccine uptake at 10 and 14 weeks of age, according to Pakistan’s EPI and the recommended schedule.

### Study Hypothesis

An Android smartphone–based personalized app will improve vaccination uptake among children at 10 and 14 weeks of age compared to standard care.

### Outcomes

#### Primary Outcomes

We aim to see the following primary outcomes: (1) a 10% increase in RCI through a personalized smartphone–based app at 10 and 14 weeks of age, according to the EPI schedule, versus standard care and (2) a 10% increase in RCI within 1 week of the original timeline at 10 and 14 weeks versus standard care.

#### Secondary Outcomes

We aim to achieve the following secondary outcomes: (1) to understand the perceptions and barriers of caregivers regarding immunization, (2) to understand caregivers’ perceptions, acceptability, and usability of a personalized mobile phone–based app for vaccination improvement, and (3) to achieve 85% accuracy in correctly predicting the likelihood of children defaulting from subsequent RCI visits.

### Study Site

This study will be conducted at the AKUH in Karachi, Pakistan. The caregivers visiting the vaccination center at the AKUH Community Health Center (CHC) will be enrolled in the study.

### Study Design

A mixed methods study will be conducted in which a smartphone app will be developed based on the findings of the qualitative component of the study, in addition to *no-show* study findings [24]. The smartphone app will include text, voice, video, and pictorial messages to help the caregiver participants adhere to their children’s RCI schedules at 10 and 14 weeks of age.

### Qualitative Component: Pretrial Interviews

The qualitative component of this study involves determining caregivers’ perceptions about RCI and the role of the mobile phone–based app in improving RCI coverage. In-depth interviews will be conducted with the caregivers visiting the CHC vaccination center. We explored the caregivers’ perceptions and attitudes regarding childhood vaccination, the reasons for missed visits at the CHC vaccination center, the perceived role of staff and services provided through the CHC vaccination center, the usability and acceptability related to the use of the personalized mobile phone–based app for improvement in vaccine uptake, the potential barriers related to the app’s usage, and their expectations of the app regarding reminders for RCI coverage and timeliness. We used the purposive sampling strategy to interview 15-20 consenting parents over 15 days. This method of sampling allows for the identification of well-informed participants who can provide insights and personal experiences regarding the subject being examined. A semistructured interview guide was developed in English and Urdu and was further modified after each pilot interview to explore the concerns recognized through constant analysis of consecutive interviews. Data collection continued until data saturation was reached. Each interview took around 30-45 minutes. Information gathered through the interviews helped us in (1) understanding the types of RCI and mobile phone app barriers perceived by caregivers, (2) designing the randomized controlled trial (RCT), and (3) developing a mobile app.

### Intervention

An Android-based mobile app is being developed. The app will have features and capacity for text, voice, pictorial, and video messages. The content of the messages will be based on the previous study that utilized automated SMS text messages and calls in various local languages to improve RCI coverage [25].

The four message domains will be educational, reminder, religious, and adverse effects. In addition, pictorial and video messages freely available through open sources focusing on immunization among children in Pakistan will be utilized.

### Development of the Smartphone App

This smartphone app, as part of the Capacity Building in Technology-Driven Innovation in Healthcare (CoNTINuE) project, will be developed on an Android platform, as 95% of smartphone users in Pakistan use Android phones [26]. Functionality validation exercises will be carried out using cognitive validation and cultural probes before using the app in this research. The app will be installed on the phones of the participants in the intervention arm by the study administrators. Once the app has launched and privacy policies have been accepted, the participants will be asked to enter information into the app, including their child’s medical record number, their child’s date of birth, their preferred mode of messages such as audio or text, their language and time preferences to receive the messages, and the barriers they face to vaccinate their child; these features will help personalize the type and content of messages they will receive. The data will be stored in local secured systems and immediately anonymized using digitally available tools to ensure privacy and avoid any potential identity breach.

**Figure 1:**
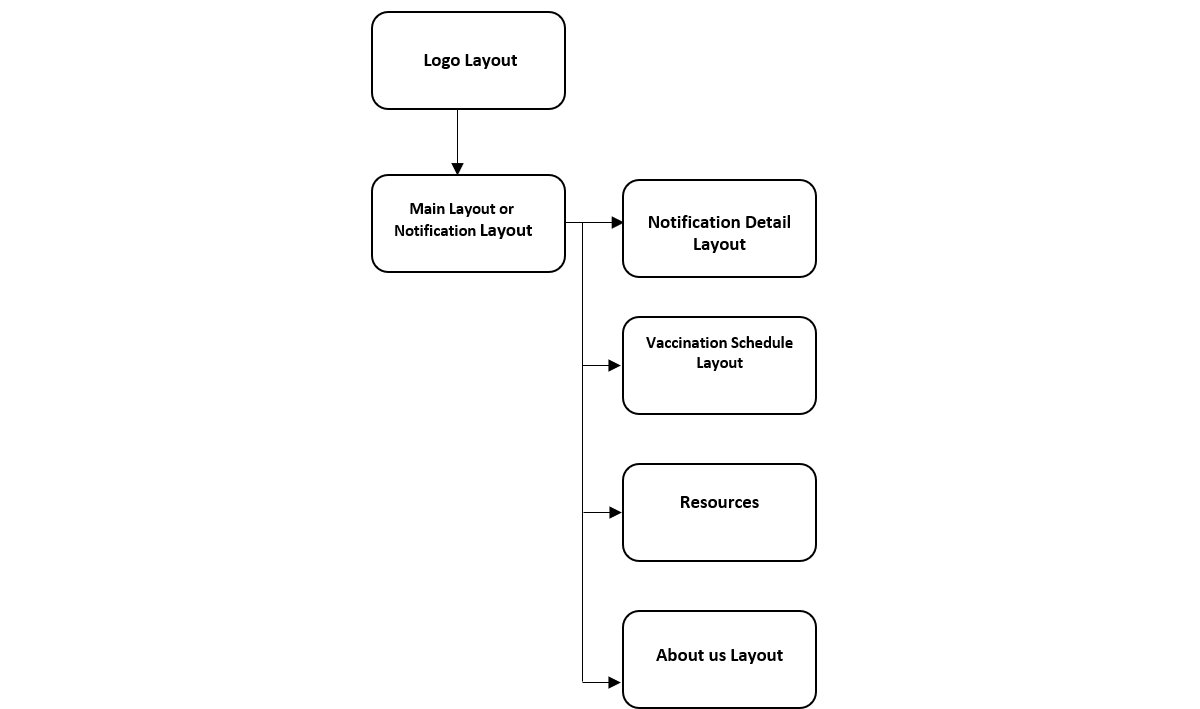
Flow diagram of the user interface.

**Figure 2:**
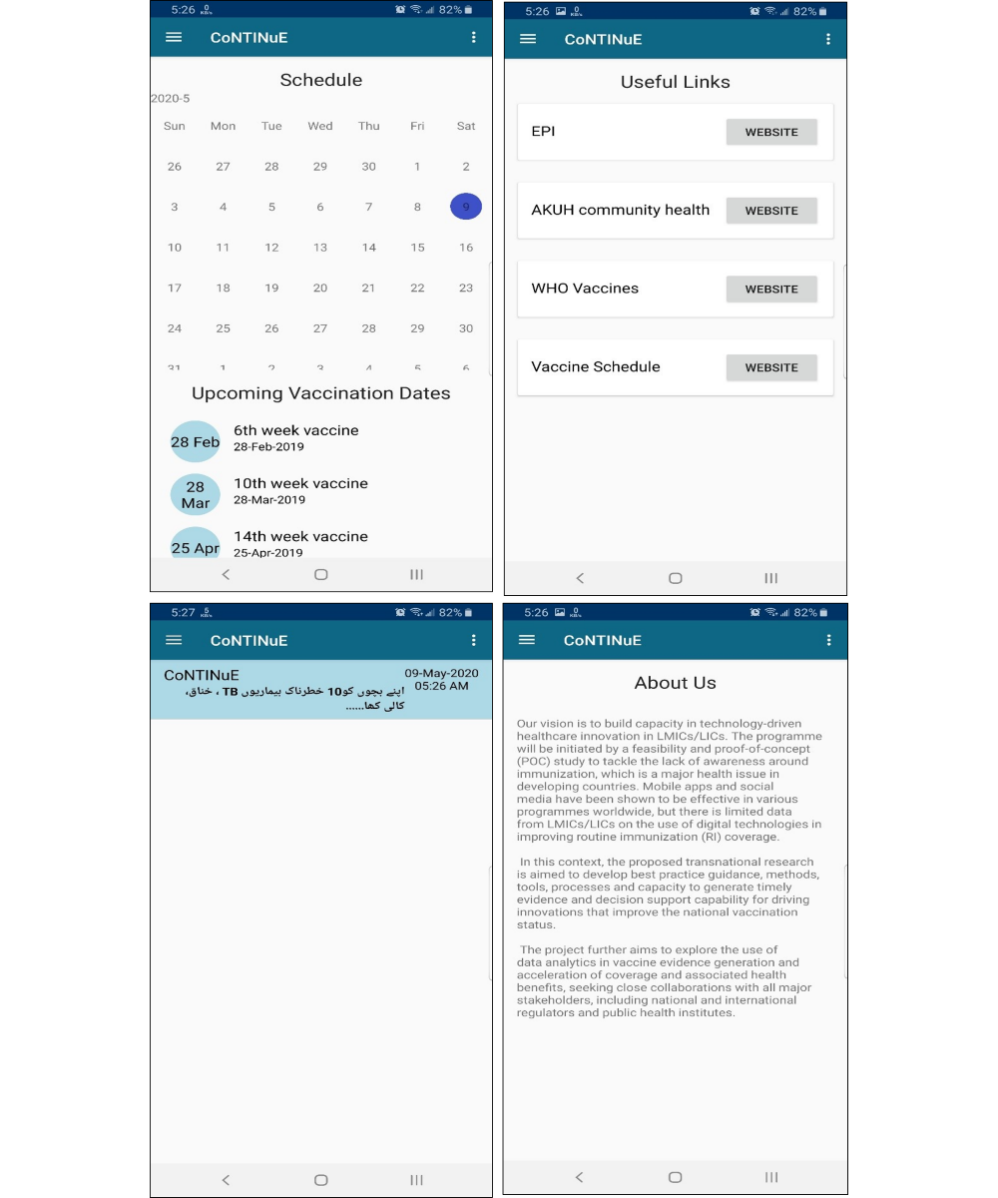
Screenshots of the various layouts of the app: vaccination schedule layout (top left), resources layout (top right), notification detail layout (bottom left), and about us layout (bottom right).

After entering the enrollment data, the phone will need to be connected to the internet for a few minutes to allow for necessary content to be downloaded, which will enable timely notifications in the future. At the enrollment site, free Wi-Fi will be available for the participants. However, in the event of a disruption in downloading the app or prolonged time consumption due to the size of the app, an alternate Wi-Fi connection, whose cost is covered by the project, will be provided to download the app. This feature of one-time downloading of the data allows for the app to be functional even in the case of internet unavailability. Once the content is downloaded, the first screen to be displayed will be the app logo layout followed by the main home layout. The user can then navigate to the (1) *vaccination schedule* layout, (2) *resources* layout, (3) *notification detail* layout, or (4) *about us* layout pages, as shown in Figures 1 and 2. This app will be developed to catalyze a behavioral change in its users through its many functionalities. In the *vaccination schedule* layout, a calendar indicating the child’s specific, scheduled vaccination days will be displayed. In the *resources* layout, the users will be able to access the official websites of the EPI, the AKUH CHC, and World Health Organization vaccines, among others. The *notification detail* layout will display notifications every week regarding upcoming vaccinations or educational messages. The notifications will be displayed as summaries, and additional information and content from the text or audio message will be able to be accessed upon clicking the individual notification. The notifications will be delivered in the participant’s preferred language (ie, Urdu, English, Sindhi, Roman Urdu, and Roman Sindhi), preferred mode of messaging (ie, text or audio), and preferred time for receiving the notifications (ie, morning, afternoon, or evening). Lastly, the *about us* layout will provide insight into the vision of the CoNTINuE project. These navigation panels and their labels will also be displayed in the preferred local language. Also, at the time of enrollment, a detailed walk-through process of the app will be conducted with the caregivers.

### Development of the AI-Enabled App

AI is being applied in this study in two different manners. First, AI is being applied to the baseline data and RCT results from a previous study, which is based on an automated SMS text message and voice call intervention [25]. Paigham-e-Sehat is a recently completed study that includes data from 3300 children, in which RCI-related messages were sent to caregivers via SMS text messages or automated calls to improve their children’s vaccine coverage at 6, 10, and 14 weeks of age. An AI model, using decision tree classification, random forest, naïve Bayes, and k-nearest neighbor algorithms, generated through that study is being used to train the system for predicting the likelihood of a family defaulting from subsequent RCI visits. This system will be tested during this study by validating the system’s prediction ability against the actual outcome of this study. Further, the important predictors and barriers identified in the previous study will be used for extending the work on data obtained from this study [25]**.** After the availability of the study results, factors related to the effect of users’ app behavior on the vaccination outcomes, including their preferred modes, timing, and interactivity with the app, will be analyzed through pattern recognition using a mixture of hidden Markov models. These findings will then be used to train the next version of the app to automatically adjust to individual user behaviors that have been found to be effective in improving RCI coverage (eg, the pattern recognition may identify a cluster of participants that have a better chance of getting their children immunized if they interact with app notifications during the weekend, when they are more likely to plan the next week).

### Trial RCT

#### Study Design, Settings, and Timeline

The study design is an RCT, consisting of two parallel arms with the allocation of 1:1 to the intervention group or the standard care group (ie, control group). After obtaining informed consent, eligible infants and their caregivers will be randomly allocated into one of two groups using randomly generated computer assignments, where allocations will be placed in sealed opaque envelopes to be opened at the time of enrollment. IDs will be generated in blocks, with 6 cases in each block. Details of each group are as follows:

1. Intervention group: the intervention group, in addition to the standard counseling group, will receive text, voice, pictorial, and video messages regarding vaccinations; messages will be received once a week until the child turns 14 weeks of age.
2. Control group: the control group will receive one-time, standard, verbal counseling at the time of the initial visit for on-time EPI vaccines at 10 and 14 weeks of age, as recommended by the EPI and the government of Pakistan.

The trial was registered at ClinicalTrials.gov (NCT04449107).

#### Inclusion and Exclusion Criteria

The study inclusion criteria will be as follows: (1) a parent or guardian, or at least one person in the household, has a working Android-based smartphone, (2) the parent or guardian has a basic ability to use an Android-based smartphone, and (3) the parent or guardian provides consent. The study exclusion criterion is that the family does not plan to visit the AKUH for scheduled CRI visits when their child is 10 and 14 weeks of age.

#### Sample Population and Size

Target enrollment is 328 infants, 164 in each arm; assumptions used for calculating sample size are an increase in RCI coverage rate from 40% to 60%, power of 0.80, α error rate of .05, and allowing for 10% dropout [27].

#### Sampling Methodology

The study staff will approach families visiting the AKUH CHC vaccination center, who have brought an infant for their 6-week RCI. Staff will explain the study objectives to the parents or caregivers. If the parent or caregiver is interested in the study, the infant will be evaluated for enrollment. One child per household will be selected. In a household where there is more than one child due for a 10- or 14-week vaccination, a random selection will be made by a program designed in the mobile phone device. After meeting the eligibility criteria, informed consent will be obtained, and the infant will be enrolled in the study.

#### Randomization

The intervention and control group ratio will be 1:1; the randomization list will be generated through computer assignments with blocks of 6 children; participant allocation will be placed in sealed opaque envelopes that will be opened at the time of enrollment after informed consent.

### Data Analysis

#### Qualitative Data

The data will be transcribed into a written form from audio recordings and will be analyzed via the qualitative data analysis software NVivo 11 (QSR International). Written transcripts will then be uploaded into NVivo 11 software to offer easy and organized retrieval of data for analysis. The data analysis will be conducted according to discourse analysis to identify the themes and subthemes conveying the underlying conscious or unconscious intent of the caregivers through language. The interview guides will also be pretested in a similar community.

#### Quantitative Data

Analyses will be conducted according to the intention-to-treat principle. The primary outcome is to assess the difference in vaccination status between the intervention and control arms. The chi-square test will first be used to compare sample characteristic proportions between the groups.

### Exit Survey

A second survey will be conducted when the child visits the CHC vaccination center for their 14-week CRI or at 18 weeks of age, whichever comes first. If the caregiver did not visit the facility at 14 weeks, they will be phoned when their child reaches 18 weeks of age to complete the exit survey in order to identify their vaccination coverage according to the schedule, to be confirmed by physical examination of their EPI card.

### Ethical Considerations

The study protocol and associated study instruments, including consent forms in English and local languages, have been approved by Aga Khan University’s Ethics Review Committee. The study will be conducted in accordance with the Declaration of Helsinki and established guidelines. All participants will provide informed consent before participation. Participants will have the right to refuse to participate in the study or leave the study at any time; this will not affect any services provided to them at the health center. Data confidentiality will be maintained at all times. No personal identifiers will be used in any reports or publications of the study.

### Data Management, Confidentiality, Privacy Protection, and Quality Assurance

The audio recording and transcripts will have unique identifiers; original and backup files will be archived in a password-protected computer system at Aga Khan University. Only transcripts and themes will be shared at NED University and the University of Surrey without nominative information. All study-related data including the recordings will be stored in an encrypted server with password protection and will only be accessible by the study-specific personnel.

Baseline and follow-up data will be collected on a smartphone device via a survey. The entry program will be designed to capture data as well as the location of the interviewer along with some monitoring parameters. Each child participant will be given a structured unique identifier. Business rules; skips, using skip logic; and consistency checks will be incorporated, and important fields will be marked as *must enter* in the questionnaire to maintain data collection quality. The database will reside on a central computer at Aga Khan University, which will be managed by the study staff. A web-based dashboard will be designed to report daily study progress. Mobile phone numbers will not be shared except to track and analyze patterns of use. Only relevant study staff will have access to study the data that has been allowed by the local ethics committee. All study staff will undergo basic research ethics training. Participants’ information will be given a study code, and no personal identifiers will be shared. Data confidentiality will be maintained at all times. No personal identifiers will be used in any reports or publications of the study. No individual identifiers, such as names of participants or their locations, will be shared. In addition, a confidentiality agreement has been signed with the universities stating that the ID numbers provided will only be used for the trial. All audio recordings will be destroyed within 5-7 years of the study, according to the recommendation of the AKUH ethics committee.

## Results

Pretrial, qualitative, in-depth interviews were conducted in February 2020. The main theme extracted was that mothers are the decision makers of the family regarding child health. The majority of the caregivers said that they preferred text messages rather than audio messages because (1) they can be stored in mobile phones and can be accessed anytime, (2) sometimes audio is not clear, and (3) audio cannot be played everywhere, but text can be read easily. There were no time preferences regarding the receipt of app notifications by the caregivers. Most of the parents and caregivers used Android-based smartphones. English and Urdu were the preferred languages for immunization-related messages. According to the caregivers, the mobile app should have information regarding the doses and purpose of all the childhood immunizations to be given until 5 years of age. In addition to immunization-related messages, the parents were also interested in monitoring their children’s health progress through the app. Parents found the idea of a mobile app helpful, as it will remind them of their children’s vaccine due dates, will provide additional information regarding immunization, and will have no installation charges.

## Discussion

### Overview

The COVID-19 pandemic and distancing measures have further drastically decreased CRI coverage in LMICs settings, mainly because caregivers avoid tertiary care hospitals or primary health centers for obtaining their children’s vaccines. This study is the first one of its kind to evaluate the efficacy of an AI-incorporated, Android-based, immunization app offering different kinds of messages—text, pictorial, and audio—in LMICs. The messages will be sent to the participants once a week. We will also assess the sharing of information and communication between the family members regarding immunization in the exit survey.

The pretrial qualitative interviews will generate insights into the perceptions and beliefs of the families regarding immunizations and their expectations of the smartphone app. This information will be extremely valuable in designing interventions that take local context and challenges into account.

The findings of this study will be useful for any future studies in Pakistan and in LMICs, in general. This information will be disseminated on a continuous basis among governmental and nongovernmental organizations, policy makers, community leaders, the telecom sector, and other stakeholders in the digital health sector in Pakistan. Study findings will be submitted for publication in a peer-reviewed journal with an international public health audience.

### Strengths

This is the first study of its kind to examine the efficacy of an Android-based mobile phone health app in improving CRI coverage in an LMICs setting. This is a mixed methods study augmented by qualitative interviews and an RCT.

### Limitations

This study will be conducted in a private, tertiary, health care hospital which may not be truly representative of the low socioeconomic status of the region’s population. The app will only be available for Android users and, therefore, iOS users will be excluded from the study; however, both control and intervention arms will only include participants with Android-based mobile phones.

## Appendix

Multimedia Appendix 1 CONSORT-eHEALTH checklist (V1.6.1).

## Abbreviations

AI: artificial intelligence
AKUH: Aga Khan University Hospital
BCG: bacillus Calmette Guérin
CHC: Community Health Center
CoNTINuE: Capacity Building in Technology-Driven Innovation in Healthcare
DTP: diphtheria-tetanus-pertussis
EPI: Expanded Programme on Immunization
LMICs: low- and middle-income countries
PDHS: Pakistan Demographic and Health Survey
RCI: routine childhood immunization
RCT: randomized controlled trial

## References

[1] Pakistan: Key demographic indicators. UNICEF Data.

[2] (2020). Children: Improving survival and well-being. World Health Organization.

[3] Butt M, Mohammed R, Butt E, Butt S, Xiang J (2020). Why have immunization efforts in Pakistan failed to achieve global standards of vaccination uptake and infectious disease control?. Risk Manag Healthc Policy.

[4] Pakistan: Expanded Programme on Immunization. World Health Organization.

[5] Imran H, Raja D, Grassly N, Wadood M, Safdar R, O'Reilly KM (2018). Routine immunization in Pakistan: Comparison of multiple data sources and identification of factors associated with vaccination. Int Health.

[6] Riaz H (2013). Public health failings behind Pakistan's measles surge. Lancet.

[7] National Institute of Population Studies (NIPS), Pakistan, and ICF (2019). Pakistan Demographic and Health Survey 2017-18.

[8] Shaikh BT, Haq ZU, Tran N, Hafeez A (2018). Health system barriers and levers in implementation of the Expanded Program on Immunization (EPI) in Pakistan: An evidence informed situation analysis. Public Health Rev.

[9] Noh J, Kim Y, Akram N, Yoo KB, Cheon J, Lee LJ, Kwon YD, Stekelenburg J (2019). Determinants of timeliness in early childhood vaccination among mothers with vaccination cards in Sindh province, Pakistan: A secondary analysis of cross-sectional survey data. BMJ Open.

[10] Zaidi SMA, Khowaja S, Kumar Dharma V, Khan AJ, Chandir S (2014). Coverage, timeliness, and determinants of immunization completion in Pakistan: Evidence from the Demographic and Health Survey (2006-07). Hum Vaccin Immunother.

[11] Haq Z, Shaikh BT, Tran N, Hafeez A, Ghaffar A (2019). System within systems: Challenges and opportunities for the Expanded Programme on Immunisation in Pakistan. Health Res Policy Syst.

[12] Kirmani S, Saleem A (2020). Impact of COVID-19 pandemic on paediatric services at a referral centre in Pakistan: Lessons from a low-income and middle-income country setting. Arch Dis Child.

[13] Telecom indicators. Pakistan Telecommunication Authority (PTA).

[14] Franzini L, Rosenthal J, King H, Balderas L, Brown M, Milne G, Drutz J, Evans D, Kozinetz C, Oettgen B, Spears W, Hanson IC (1999). Cost and effectiveness of immunization reminder/recall systems for private providers. Pediatr Res.

[15] Stockwell MS, Fiks AG (2013). Utilizing health information technology to improve vaccine communication and coverage. Hum Vaccin Immunother.

[16] Jacobson VJ, Jacobson R, Coyne-Beasley T, Asafu-Adjei J, Szilagyi P (2018). Patient reminder and recall interventions to improve immunization rates. Cochrane Database Syst Rev.

[17] Oliver-Williams C, Brown E, Devereux S, Fairhead C, Holeman I (2017). Using mobile phones to improve vaccination uptake in 21 low- and middle-income countries: Systematic review. JMIR Mhealth Uhealth.

[18] Domek GJ, Contreras-Roldan IL, O'Leary ST, Bull S, Furniss A, Kempe A, Asturias EJ (2016). SMS text message reminders to improve infant vaccination coverage in Guatemala: A pilot randomized controlled trial. Vaccine.

[19] Eze GU, Adeleye OO (2015). Enhancing routine immunization performance using innovative technology in an urban area of Nigeria. West Afr J Med.

[20] Poorman E, Gazmararian J, Parker RM, Yang B, Elon L (2015). Use of text messaging for maternal and infant health: A systematic review of the literature. Matern Child Health J.

[21] Bangure D, Chirundu D, Gombe N, Marufu T, Mandozana G, Tshimanga M, Takundwa L (2015). Effectiveness of short message services reminder on childhood immunization programme in Kadoma, Zimbabwe - A randomized controlled trial, 2013. BMC Public Health.

[22] Stockwell MS, Kharbanda EO, Martinez RA, Lara M, Vawdrey D, Natarajan K, Rickert VI (2012). Text4Health: Impact of text message reminder-recalls for pediatric and adolescent immunizations. Am J Public Health.

[23] Stockwell MS, Hofstetter AM, DuRivage N, Barrett A, Fernandez N, Vargas CY, Camargo S (2015). Text message reminders for second dose of influenza vaccine: A randomized controlled trial. Pediatrics.

[24] Saeed S, Somani N, Sharif F, Kazi AM (2018). Evaluating the effectiveness of text messaging and phone call reminders to minimize no show at pediatric outpatient clinics in Pakistan: Protocol for a mixed-methods study. JMIR Res Protoc.

[25] Kazi AM, Ahsan N, Khan A, Jamal S, Kalimuddin H, Ghulamhussain N, Wajidali Z, Muqeet A, Zaidi F, Subzlani M, McKellin W, Ali A, Collet J (2019). Personalized text messages and automated calls for improving vaccine coverage among children in Pakistan: Protocol for a community-based cluster randomized clinical trial. JMIR Res Protoc.

[26] Mobile operating system market share Pakistan. StatCounter.

[27] Kazi AM, Ali M, Zubair K, Kalimuddin H, Kazi AN, Iqbal SP, Collet J, Ali SA (2018). Effect of mobile phone text message reminders on routine immunization uptake in Pakistan: Randomized controlled trial. JMIR Public Health Surveill.

